# Graphene Oxide-Based Nanocomposites Decorated with Silver Nanoparticles as an Antibacterial Agent

**DOI:** 10.1186/s11671-018-2533-2

**Published:** 2018-04-23

**Authors:** Sławomir Jaworski, Mateusz Wierzbicki, Ewa Sawosz, Anna Jung, Grzegorz Gielerak, Joanna Biernat, Henryk Jaremek, Witold Łojkowski, Bartosz Woźniak, Jacek Wojnarowicz, Leszek Stobiński, Artur Małolepszy, Marta Mazurkiewicz-Pawlicka, Maciej Łojkowski, Natalia Kurantowicz, André Chwalibog

**Affiliations:** 1 Division of Nanobiotechnology, Warsaw University of Life Science, Ciszewskiego 8, 02-786 Warsaw, Poland; 2 Military Institute of Medicine, Szaserów 128, 04-141 Warsaw, Poland; 3 Braster S.A., Cichy Ogród 7, 05-580 Ożarów Mazowiecki, Poland; 4 Faculty of Mechatronics, Warsaw University of Technology, Boboli 8, 02-525 Warsaw, Poland; 5 Institute of High Pressure Physics of the Polish Academy of Sciences, Sokołowska 29/37, 01-142 Warsaw, Poland; 6 Faculty of Chemical and Process Engineering, Warsaw University of Technology, Waryńskiego 1, 00-645 Warsaw, Poland; 7 Faculty of Materials Science and Engineering, Warsaw University of Technology, Pl. Politechniki 1, 00-661 Warsaw, Poland; 8 Department of Veterinary and Animal Sciences, University of Copenhagen, Groennegaardsvej 3, 1870 Frederiksberg, Denmark

**Keywords:** Graphene oxide, Silver nanoparticles, Antimicrobial properties

## Abstract

One of the most promising methods against drug-resistant bacteria can be surface-modified materials with biocidal nanoparticles and nanocomposites. Herein, we present a nanocomposite with silver nanoparticles (Ag-NPs) on the surface of graphene oxide (GO) as a novel multifunctional antibacterial and antifungal material. Ultrasonic technologies have been used as an effective method of coating polyurethane foils. Toxicity on gram-negative bacteria (*Escherichia coli*), gram-positive bacteria (*Staphylococcus aureus* and *Staphylococcus epidermidis*), and pathogenic yeast (*Candida albicans*) was evaluated by analysis of cell morphology, assessment of cell viability using the PrestoBlue assay, analysis of cell membrane integrity using the lactate dehydrogenase assay, and reactive oxygen species production. Compared to Ag-NPs and GO, which have been widely used as antibacterial agents, our nanocomposite shows much higher antimicrobial efficiency toward bacteria and yeast cells.

## Background

The development of antibiotics has played a significant role in controlling the number of bacterial infections. However, the improper use and the overuse of antibiotics have led to the development of multidrug resistance in many bacterial species. Some strains have become resistant to practically all of the commonly available agents: beta-lactams, tetracyclines, and aminoglycosides [1]. The major resistant pathogens are methicillin-resistant *Staphylococcus aureus*, vancomycin-resistant *Enterococcus*, and extended-spectrum β-lactamase-producing *Klebsiella pneumoniae* and *Escherichia coli* [2, 3]. Bacteria, with their very large populations and fast proliferation time, are able to rapidly develop mechanisms of antibiotic resistance when a subset of the bacteria population survives antibiotic treatment. Moreover, antibiotic-resistant bacteria are able to transfer copies of DNA that code for a mechanism of resistance to other distantly related bacteria, which are then able to pass on the resistance genes to subsequent generations. Thus, the emergence of antibiotic-resistant bacteria represents a serious problem that could be overcome by the development of novel antimicrobial agents. Antibacterial agents are very important in the textile industry, water disinfection, medicine, and food packaging. Nanoparticles and nanomaterials can be used as an alternative to antibiotics [4]. The mechanism of antibacterial activity of nanoparticles varies among the different types of nanoparticle. While some proposed mechanisms relate to the physiochemical structure of the nanoparticles, others relate to the increased release of antibacterial ions from nanoparticle surfaces. Multiple simultaneous mechanisms of action against microbes would require a variety of synchronous DNA mutations in the same microbial cell for the development of resistance; therefore, it is difficult for bacterial cells to become resistant to nanoparticles and nanomaterials. Antimicrobial nanomaterials, such as silver, copper, fullerenes, and single-walled carbon nanotubes, may offer several advantages due to their unique physicochemical properties and high surface areas [5–8]. The exact mechanisms of nanoparticle (NP) toxicity against various bacteria are not completely understood. According to the current research, the major processes underlying the antibacterial effects of NPs are disruption of the bacterial cell membrane, metal ion release, generation of ROS, penetration of the bacterial cell membrane, and induction of intracellular antibacterial effects, including interactions with DNA and proteins [9, 10]. NPs are able to attach to the membrane of bacteria by electrostatic interaction and disrupt the integrity of the bacterial membrane. The positive charge of the surface of the NPs is essential for the adhesion. The positive charge enables electrostatic addition between NPs and negatively charged cell membrane of the microorganisms [11]. The electrostatic connection between NPs with the sulfur-containing proteins present on the surface of bacterial cells causes irreversible changes in cell wall structure resulting in damages of cell wall and membrane [12]. The bacterial membrane is crucial, irrespective of the metabolic status of the cell, as it provides selective permeability for cellular homeostasis and metabolic energy transduction. The second antibacterial and antifungal activity of NPs is due to their ability to produce ROS and free radical species [13]. Increased level of ROS induced hyperoxidation of lipids, proteins, and DNA [14].

Moreover, the structures of many types of NPs are suitable for carrying antimicrobial agents [15, 16]. Carriers can help to protect the drugs from resistance by target bacteria. A nanoparticle-based drug delivery system can help to target antibiotics to an infection site and thereby minimize systemic side effects. Other advantages include improved solubility of hydrophobic drugs, prolonged systemic circulation time and drug half-life, and sustained drug release [4].

Recently, it has been demonstrated that graphene, a new allotrope of carbon, has antibacterial activity. Graphene is a material made of carbon atoms that are bonded together in a repeating pattern of hexagons. A unique feature of graphene flakes is the ratio of its thickness to the surface. The surface of graphene is covered with an electron cloud, which probably predisposes this material to be an electron donor and gives it the ability to make special bonds. The edges of graphene have other bonds (characteristic for diamond sp3 type bonds), and these places may have different physicochemical characteristics [17]. These characteristics suggest that graphene can be exposed to plastic adhesion to different intercellular structures, including bacterial cells [18–20]. In addition, because it has two active sides (surface and edges), graphene can attach biological molecules to its edges and adhere to the cell surface. An oxidized form of graphene, graphene oxide (GO), is easily dispersible in water and other organic solvents due to the presence of the oxygen functionalities. The oxygenated groups enable the straightforward chemical functionalization of GO sheets via covalent and non-covalent interactions. The strong antibacterial activity of GO has been reported. The antibacterial activity of GO has been assigned to membrane stress induced by sharp edges of graphene oxide nanosheets, which may result in physical damage to cell membranes, leading to the loss of bacterial membrane integrity [21]. Recently, graphene-functionalized antimicrobial nanoparticles have been used as promising antibacterial materials [22, 23]. Nanocomposites can overcome the limitations of the individual components. For example, antibacterial nanomaterials attached to the graphene substrate are more stable and well dispersed [24]. These nanocomposites could contain metals, metal oxides, and polymers.

One of the most promising methods against drug-resistant bacteria can be surface-modified materials with biocidal nanoparticles. Ultrasonic technologies have been confirmed as an effective method of coating various materials with antibacterial and fungicidal substances [25–28]. Many researchers classify the ultrasound method as a “green technology” [29, 30]. The method is based on the use of cavitation phenomena, which is the formation, growth, and collapse of cavitation bubbles in the liquid medium [31, 32]. Imploding bubbles generate immense amounts of energy in microregions up to 5000 K and pressure up to 2000 atm within a short period of time [33, 34]. Consequently, shock waves and so-called microjets directed toward the solid surface are generated [35]. Located in a liquid medium, NPs are driven up by the implosion effect and jet streams at high speed (> 100 m/s) on the solid surface and form a layer [36]. Acoustic cavitation can also lead to change in the physical properties of sonicated objects, e.g., resizing of GO flakes [37, 38]**.**

We achieved promising results in our previous studies with *Salmonella enterica* and *Listeria monocytogenes* treated with pristine graphene, GO, and reduced GO [20]. Of the different types of graphene, GO was also found to have the highest antibacterial activity at a low concentration. Bacterial cells were distributed over the entire surface of the GO. In this study, we hypothesized that GO decorated with silver nanoparticles (GO-Ag) will have stronger toxic influence on microbial cells than bare GO or silver nanoparticles (Ag-NPs). Because it has two active sides (surface and edges), GO oxide can attach Ag-NPs to the edges and adhere to the cell surface. The antibacterial activity of graphene-based nanocomposites may be due to the disruption of the cell membrane and oxidative stress. The objective of this study was to evaluate the antimicrobial activity of GO-based nanocomposites decorated with Ag-NPs in comparison to bare GO and Ag-NPs using gram-negative bacteria (*Escherichia coli*), gram-positive bacteria (*Staphylococcus aureus* and *Staphylococcus epidermidis*), and pathogenic yeast (*Candida albicans*) using an in vitro model. The investigation consisted of structural analysis of nanocomposites using X-ray diffraction, Raman spectroscopy transmission, FT-IR, electron microscopy (TEM), scanning electron microscopy (SEM) and atomic force microscopy (AFM), evaluation of microbial cell morphology, assessment of cell viability by PrestoBlue™ assay, investigation of cell membrane integrity by lactate dehydrogenase assay (LDH), and assessment of reactive oxygen species (ROS) production.

## Methods

### Synthesis, Modification, and Characterization of Graphene Oxide

In this study, a commercially available graphite powder (Acros Organics, New Jersey, USA) was oxidized by the modified Hummers method [39]. Ten grams of graphite powder were mixed with 230 mL of concentrated sulfuric acid (98%) below 10 °C. Then, 4.7 g of sodium nitrate and 30 g of potassium permanganate were added gradually to the sulfuric acid and graphite mixture while maintaining the temperature below 10 °C. Then, the mixture was heated to 30 °C and stirred for 2 h. In the next step, 100 mL of water was added, and the mixture temperature reached ~ 100 °C. Finally, the mixture was treated with 10 mL of hydrogen peroxide. For purification, the slurry was filtrated and washed with deionized water until the pH of the filtrate reached 6.5.

X-ray diffraction patterns of GO were gathered at room temperature within the range of 2 theta angle from 10° to 100° with the step of 0.02° using the X-ray powder diffractometer (*CuK*_*α1*_) (X’Pert PRO, PANalytical, Almelo, Netherlands).

The analysis of carbon, hydrogen, nitrogen, and sulfur content by weight in GO was carried out using the Vario EL III apparatus produced by Elementar Analysensysteme GmbH (Langenselbold, Germany). Prior to performing measurements of chemical analyses of GO, the samples were subject to 24-h desorption in a desorption station (VcPrep 061, Micromeritics, Norcross, GA, USA) under vacuum (0.05 mbar) at 50 °C. Oxygen content was calculated by subtracting the determined contents of carbon, hydrogen, nitrogen, and sulfur from 100% weight.

Raman spectroscopy was performed using an inVia Raman microscope (Renishaw, UK). Graphene oxide was analyzed with the 514-nm laser wavelength with the 5% of its initial power. The spectra were collected from five different spots on the sample. The exposure time was 10 s and two scans were collected.

FT-IR measurements were performed using Nicolet iS10 spectrometer (Thermo Fisher Scientific, USA) in attenuated total reflectance mode on a diamond crystal. Five microliters of graphene oxide water suspension was dripped on the surface of the diamond crystal and it was left to dry. After it was dried, the spectrum was collected in the range 400–4000 cm^−1^.

Average particle size and zeta potential measurements were carried out using Zetasizer Nano-ZS ZEN 3600 produced by Malvern Instruments Ltd. (Malvern, UK) using the dynamic light scattering (DLS) mode and laser Doppler electrophoresis, respectively, at room temperature (23 °C).

### TEM/SEM/AFM Analysis of Nanomaterials

The morphology of powders and foils was determined using the transmission electron microscope (TEM; JEM-1220 JEOL, Tokyo, Japan, accelerating voltage of 80 kV) and scanning electron microscope (SEM; Zeiss, Ultra Plus, Oberkochen, Germany). Samples for TEM observations were prepared by placing droplets of hydrocolloids onto TEM grids (Formvar on 3 mm 200 Mesh Cu Grids, Agar Scientific, Stansted, UK). Immediately after air-drying the droplets, the grids were inserted into the microscope.

For SEM analysis, samples were coated with a thin carbon layer using the sputter coater (SCD 005/CEA 035, BAL-TEC, Pfäffikon, Switzerland). An internal laboratory measurement procedure was applied (P5.10, edition 6 of 26.08.2015).

AFM (atomic force microscopy) imaging was carried out using Asylum Research MFP3D Bio software (version: Asylum Research MFP3D 15.106.09). Surface topography imaging and detection of GO on the tested foil surfaces were carried out using two imaging modes, AC mode for phase contrast imaging and lateral force microscopy (LFM) for GO detection since GO reduces friction forces [40]**.**

### Preparation of Polyurethane Foils Coated with GO and Ag-NPs

For covering polyurethane foils, suspensions of Ag-NPs (HydroSilver1000, Amepox, Łódź, Poland) and GO were used. Suspensions of GO, Ag-NPs, and GO-Ag (GO (200 μg/mL), Ag-NPs (100 μg/mL), GO (200 μg/mL) + Ag-NPs (100 μg/mL)) were prepared in deionized water (conductance 0.09 μS/cm, deionizer: HLP 20UV, Hydrolab, Staszyn, Poland). The suspensions were used without additional purification and filtration.

Ultrasonic coating of polyurethane foils (15 × 15 × 0.05 mm) took place in a glass flask with a volume of 50 ml. Foil samples were fastened on a stand (Teflon) and subsequently immersed in the prepared suspensions. The coating process was performed using an ultrasonic horn (Ti horn, Ø13 mm, 60% efficiency, 20 kHz, Sonics & Materials, Inc., Newtown, CT, USA) placed square to the foil samples present in the suspension. The process temperature was 30 ± 1 °C. The covered samples were flushed in deionized water and dried in a laminar chamber and subsequently packed in sterile packages.

### Surface Free Energy

Wettability tests were carried out using the Data Physics OCA – 20 goniometer (DataPhysics Instruments GmbH, Filderstadt, Germany). Surface free energy (SFE) was calculated using the Owens, Wendt, Rabel, and Kaelble (OWRK) method using two test liquids: deionized water and diiodomethane [41].

### Bacterial and Yeast Cultivation and Preparation

*Staphylococcus aureus* (ATCC 25923) and *Staphylococcus epidermidis* (ATCC 14990), *Escherichia coli* (ATCC 25922), and *Candida albicans* (90028) were obtained from LGC Standards (Lomianki, Poland). The strains were stored as spore suspensions in 20% (*v*/*v*) glycerol at − 20 °C. Prior to their use in experiments, the strains were defrosted and the glycerol was removed by washing the bacterial cells with distilled water. The bacteria and yeast were then grown on the following nutrient media: tryptic soy agar for *S*. *aureus* and *E*. *coli*, brain heart agar for *S*. *epidermidis*, and Sabouraud’s agar for *C*. *albicans* (Merck Millipore, Darmstadt, Germany). The bacteria and yeast grown on agar plates were harvested by gently washing the plates with sterile distilled saline solution. To calculate the number of bacteria in the cell suspension, the optical density of the suspensions at 600 nm (OD_600_) was measured using a spectrophotometer (Helios Epsilon, Unicam, Milwaukee, WI, USA). Calibration curves for each of the microorganisms were prepared by performing serial tenfold dilutions (up to 10^− 5^) of bacterial and yeast suspensions of known optical density. One milliliter of each dilution was spread on petri dishes containing the nutrient medium. After 24 h of incubation at 37 °C, the number of colonies formed on the petri dishes was enumerated. Based on the results of the enumerations (conducted in triplicate), the density of the original bacterial suspension in colony-forming units (CFU)/mL was calculated.

### Antimicrobial Assay

The inoculum for the antibacterial assay was prepared from actively growing organisms (logarithmic phase). The inoculums of all microorganisms were prepared from an overnight culture grown aerobically in Mueller–Hinton (MH) broth at 37 °C. The bacterial and yeast concentration was determined by measuring optical density at 600 nm (OD_600_). Briefly, bacterial and yeast suspensions were prepared from overnight cultures and adjusted to 10^6^ CFU/ml. Inoculum was inoculated evenly onto the surface of MH agar in petri dishes by swabbing. Sterile foils coated with GO, Ag-NPs, and GO-Ag were deposited onto the agar surface. Foils without nanoparticles were used as control group. The bacteria and yeast growth under the foils was measured after 24 h of incubation at 37 °C.

### Viability Assay

Cell viability was evaluated using the PrestoBlue™ Cell Viability Assay (Life Technologies, Taastrup, Denmark). PrestoBlue™ reagent is quickly reduced by metabolically active cells, providing a quantitative measure of viability and cytotoxicity. Bacterial and yeast cells were cultured onto foils coated with GO, Ag-NPs, and GO-Ag located on inserts inserted into 6-well plates (200 μL MH broth with 5 × 10^3^ cells per foil) and incubated for 24 h. In the next step, 90 μL of each sample was transferred to 96-well plates and 10 μL of PrestoBlue™ reagent was added to each well and incubated for an additional 2 h at 37 °C. The optical density of each well was recorded at 570 nm on an enzyme-linked immunosorbent assay (ELISA) reader (Infinite M200, Tecan, Durham, NC, USA). Cell viability was expressed as the percentage (OD_test_ − OD_blank_)/(OD_control_ − OD_blank_)×100%, where OD_test_ is the optical density of cells exposed to tested foils, OD_control_ is the optical density of the control sample, and OD_blank_ is the optical density of wells without bacterial and yeast cells.

### Membrane Integrity

An LDH test (In Vitro Toxicology Assay Kit, lactic dehydrogenase based, Sigma-Aldrich, Hamburg, Germany) was used to evaluate cell membrane integrity. The resulting reduced NAD (NADH^+^) was utilized in the stoichiometric conversion of a tetrazolium dye. When cell-free aliquots of the medium from cultures were assayed, the amount of LDH activity could be used as an indicator of membrane integrity. If the membrane was damaged, intracellular LDH molecules were released into the culture medium. Bacterial and yeast cells were cultured on foils (GO, Ag-NPs, and GO-Ag) located on inserts inserted in 6-well plates (200 μL MH broth with 5 × 10^3^ cells per foil) and incubated for 24 h. Cells cultured on foil without nanoparticles were used as a control. After this time, the samples were transferred to microcentrifuge tubes and centrifuged at 1200 rpm for 5 min. One hundred microliters of supernatant were transferred to 96-well plates, and 100 μL of the LDH assay mixture was added to each well. The plate was covered and incubated for 30 min at room temperature. The optical density of each well was recorded at 450 nm on an ELISA reader (Infinite M200, Tecan, Männedorf, Switzerland). LDH leakage was expressed as the percentage {(OD_test_ − OD_blank_) − (OD_control_ − OD_blank_)/(OD_control_ − OD_blank_)}×100%, where OD_test_ is the optical density of cells exposed to tested foils, OD_control_ is the optical density of the control sample, and OD_blank_ is the optical density of wells without cells.

### SEM Analysis of Microorganisms

Prior to SEM analysis, samples of bacteria and yeast incubated on foils with GO-Ag and untreated bacteria were prepared. Briefly, a drop of bacterial and yeast culture (10^6^ CFU/ml) was incubated on foils with GO-Ag nanocomposite, or untreated bacteria was deposited on the surface of a sterile cover glass and incubated for 24 h at 37 °C inside an empty petri dish. All samples were dried and covered with gold. Finally, the samples were imaged with SEM (FEI Quanta 200, Tokyo, Japan) at an acceleration voltage of 15 kV.

### ROS Production

ROS production was evaluated using DCFDA, Cellular Reactive Oxygen Species Detection Assay Kit (Abcam, Cambridge, UK). DCFDA uses the cell permeant reagent 2′,7′–dichlorofluorescein diacetate, a fluorogenic dye that measures hydroxyl, peroxyl, and other ROS activities within the cell. After diffusion into the cell, DCFDA is deacetylated by cellular esterases to a non-fluorescent compound, which is later oxidized by ROS into 2′,7′–dichlorofluorescein (DCF). Bacterial and yeast cells were cultured on foils (GO, Ag-NPs, and GO-Ag) located on inserts inserted in 6-well plates (200 μL MH broth with 5 × 10^3^ cells per foil) and incubated for 24 h. Cells cultured on foil without nanoparticles were used as a control. After this time, the samples were transferred to microcentrifuge tubes and centrifuged at 1200 rpm for 5 min. One hundred microliters of supernatant were transferred to 96-well plates, and 100 μL of diluted DCFDA was added to each well and incubated for an additional 45 min at 37 °C in the dark. DCF production was measured by fluorescence spectroscopy with an excitation wavelength at 485 nm and an emission wavelength at 535 nm on an ELISA reader (Infinite M200, Tecan, Durham, NC, USA).

## Results

### Characteristics of GO and Ag-NPs

The chemical analysis revealed the presence of nitrogen, carbon, sulfur, hydrogen, and oxygen (Table 1).

**Table 1:** Results of chemical analyses of graphene oxide samples

Sample	Element (% wt)				
	N	C	S	H	O
Graphene oxide	0.042	48.41	0.390	1.963	49.195

**Fig. 1:**
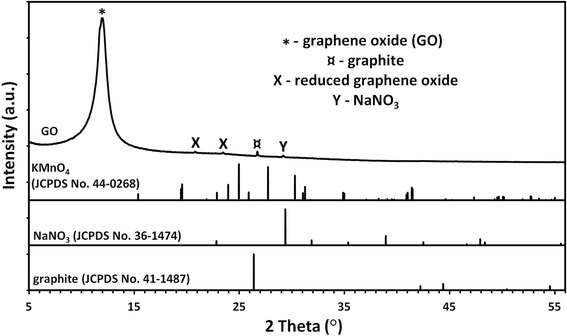
X-Ray diffraction patterns of GO powders. The phase analysis of the GO sample revealed the presence of impurities coming from trace quantities of graphite, sodium nitrate, and probably a reduced form of graphene oxide

The phase analysis of the GO sample (Fig. 1) revealed the presence of impurities coming from trace quantities of graphite, sodium nitrate, and probably a reduced form of graphene oxide.

**Fig. 2:**
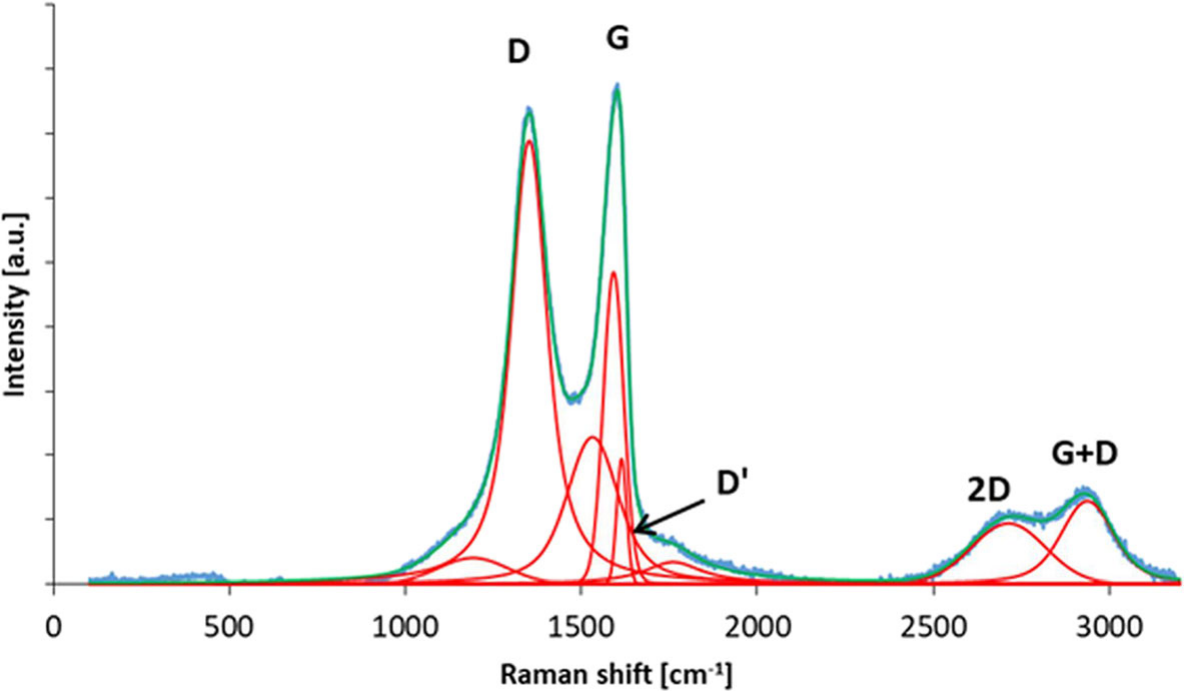
Raman spectrum of graphene oxide with proposed deconvolution of the D, G, D′, 2D, and D + G bands. GO has a highly disordered structure due to many functional groups in the structure formed during oxidation of graphite powder. The position of the D band is 1351 cm^−1^ and the G band 1590 cm^−1^; the *I*_D_/*I*_G_ ratio is 1.15

Raman spectroscopy can give information about the structural features of graphene oxide. The D band is attributed to the structural disorder, while the G band comes from the bond stretching of carbon sp^2^ atoms [42]. The additional bands (including D′, 2D, and D + G) arise from the defects present in the graphitic structure of the carbon material. *I*_D_/*I*_G_ ratio (calculated from the intensity of D and G bands) can be used to characterize the disorder of the graphitic structure in carbon materials. As can be seen in Fig. 2, GO has a highly disordered structure due to many functional groups in the structure formed during oxidation of graphite powder. The position of the D band is 1351 cm^−1^ and the G band 1590 cm^−1^; the *I*_D_/*I*_G_ ratio is 1.15.

**Fig. 3:**
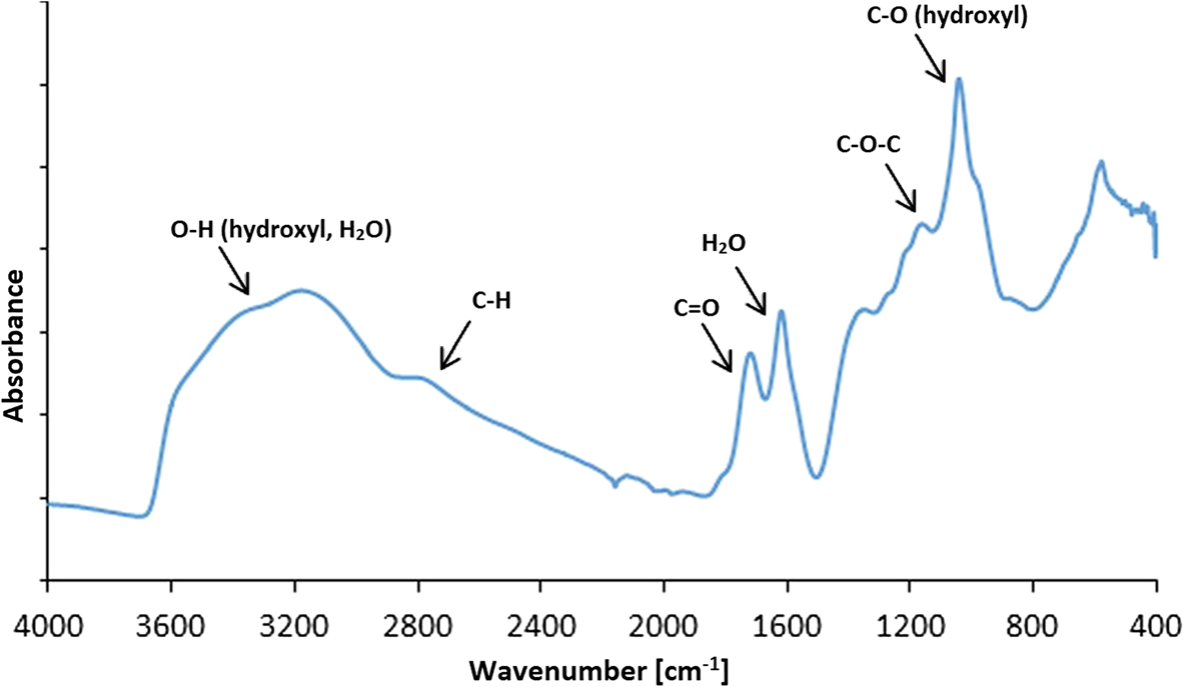
FT-IR (ATR, attenuated total reflectance) spectrum of graphene oxide with proposed assignment of functional groups present in GO. The most notable peaks were observed at ~ 3500 cm^−1^, (attributed to water and hydroxyl groups), ~ 1080 cm^−1^ (hydroxyl groups), ~ 1600 cm^−1^ (assigned to C=C bonds present in graphitic carbon). Other peaks observed on the FT-IR spectrum show that GO is rich in groups containing C=O (mainly carboxyl groups), peak around 1720 cm^−1^, epoxy (C–O–C) with the visible peak around 1200 cm^−1^, and C–H bonds (peak around 2800 cm^−1^)

**Fig. 4:**
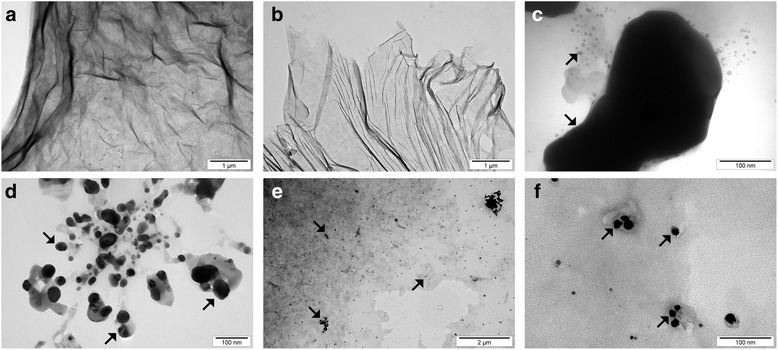
TEM images of agglomerated GO flakes (**a**), GO flakes after ultrasonic treatment (**b**), agglomerated Ag-NPs (**c**), Ag-NPs after ultrasonic treatment (**d**), and GO-Ag (**e**, **f**). The decrease of the average GO particle size after ultrasonic treatment was caused by defragmentation or folding of the GO flakes. The decrease of the average Ag size after ultrasonic treatment was caused by defragmentation of Ag agglomerates. Note: Arrows point to Ag-NPs/agglomerates

**Fig. 5:**
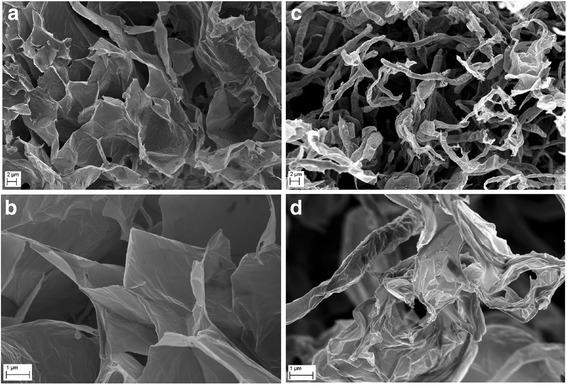
Comparison of morphology of lyophilized GO flakes (**a**, **b**) and GO flakes after ultrasonic treatment (**c**, **d**) using scanning electron microscopy. The decrease of the average GO particle size after ultrasonic treatment was caused by defragmentation or folding of the GO flakes

**Fig. 6:**
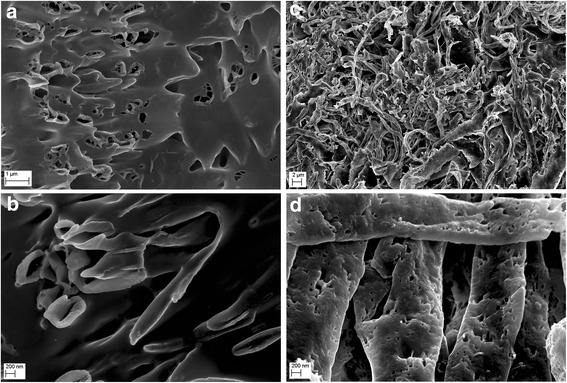
Comparison of morphology of lyophilized Ag-NP mixture (**a**, **b**) and Ag-NP mixture after ultrasonic treatment (**c**, **d**) using scanning electron microscopy

The FT-IR spectrum of graphene oxide collected in the ATR mode revealed that GO has a lot of functional groups present in the structure. The most notable peak can be observed at ~ 3500 cm^−1^, which is assigned mainly to water and hydroxyl groups (Fig. 3). A very intensive peak around 1080 cm^−1^ can also be attributed to hydroxyl groups. The peak around 1600 cm^−1^ usually is assigned to C=C bonds present in graphitic carbon. However, our previous XPS studies show that there is a few C=C bonds in graphene oxide [43]; hence, we attribute this peak to mostly water still present in the graphene oxide. Other peaks observed on the FT-IR spectrum show that GO is rich in groups containing C=O bonds (mainly carboxyl groups), peak around 1720 cm^−1^, epoxy (C–O–C) with the visible peak around 1200 cm^−1^, and C–H bonds (peak around 2800 cm^−1^). The FT-IR analysis is in good agreement with XPS measurements performed for graphene oxide where also hydroxyl, carboxyl, epoxy, and carbonyl groups were identified [44]. GO and GO after a 10-min ultrasonic homogenization were compared (Figs. 4 and 5), and similarly, Ag-NPs with Ag-NPs after a 10-min ultrasonic homogenization (Figs. 4 and 6) were compared. In order to avoid changes to compound morphology, all compounds were rapidly cooled down with liquid nitrogen and dried in a lyophilizer. Figure 5a, b presents GO flakes, while Fig. 5c, d shows the effects of ultrasounds on GO flakes, which undergo partial folding and fragmentation. Figure 6 also shows a similar effect for Ag-NPs, where a change of material morphology is visible. Figure 6a, b displays dried poly(vinyl alcohol), which was used for stabilizing the water suspension of Ag-NPs. The destructive effect of ultrasonic homogenization was notable as the polyvinyl alcohol structure was broken down into long heterogeneous parts with small spherical openings (Fig. 6c, d).

### Average Size and Zeta Potential

The results of average particle/agglomerate size in water suspensions are presented in Table 2. Analyses of average size were carried out for concentrated suspensions which were not subject to ultrasonic homogenization (as received) and for diluted suspensions. Diluted suspensions before the test were subject to ultrasonic homogenization, with homogenization parameters identical to those used during the ultrasonic coating of the foil with nanomaterial layers (Ag-NPs, GO). For Ag-NP suspension, the action of the ultrasounds caused an increase in the average particle size from 80 to 218 nm. The main cause of the increase in the average particle size after ultrasonic homogenization in the suspension (apart from the process of Ag-NP agglomeration) was that Ag-NPs were driven into the poly(vinyl alcohol) that was used for suspension stabilization. The large standard deviation of the Ag-NP sample homogenized by ultrasound resulted from the presence of both loose Ag-NPs and Ag-NPs driven into the poly(vinyl alcohol) in the suspension. In the case of GO suspension, the average particle size of the sample subject to ultrasonic homogenization was 263 nm and was ca 7.7 times smaller than the average particle size of the sample that was not subject to homogenization. The obtained results are convergent with the SEM tests (Fig. 5), which show the destructive effect of ultrasounds on GO flakes. The decrease of the average GO particle size was caused by defragmentation or folding of the GO flakes. However, it should be emphasized that the results of the average particle size of GO suspension samples involve an error related to the nanomaterial shape. The results obtained by the DLS method are a hydrodynamic average that is calculated based on the shape of a sphere that has the same diffusion coefficient as the measured particles; however, the shape of GO was flakes, which was confirmed by SEM images.

**Table 2:** Test results of average particle/agglomerate size in suspensions

Sample	Size by DLS, Z-average, diameter [nm] ± SD
Ag-NPs (as received)	80 ± 1
Ag-NPs (100 μg/mL, after US)	218 ± 93
GO (as received)	2030 ± 36
GO (200 μg/mL, after US)	263 ± 8
GO (200 μg/mL)-Ag (100 μg/mL)	251 ± 10

*DLS* dynamic light scattering, *Z-average* harmonic intensity averaged particle diameter, *SD* standard deviation, *US* ultrasound method, *Ag-NPs (as received)* HydroSilver1000 (Amepox, Łódź, Poland) material was not processed Ag-NPs (100 μg/mL, after US) as above but was processed under US under similar conditions to the foils in the preceding sections, *GO (as received)* material not processed, *GO (200 μg/mL, after US)* as above but processed under US under similar conditions to the foils in the preceding sections, *GO (200 μg/mL)-Ag (100 μg/mL)* materials mixed together and processed under US under similar conditions to the foils in the preceding sections

Test results of the zeta potential analysis of samples are provided in Table 3. The zeta potential of Ag-NPs in a water suspension was merely − 5.9 mV, which resulted in a lack of electrostatic stability of the sample. The sample of Ag-NP suspension was stabilized sterically by preserving Ag-NP distances through poly(vinyl alcohol) addition, which prevented agglomeration/aggregation of Ag-NPs. The zeta potential of the GO suspension sample, in turn, was − 41 mV, which gave a moderate electrostatic stability to the sample. A moderate electrostatic stability of a sample is characterized by slow sedimentation with virtually negligible change of particle size in the period of declared fitness of the suspension. The zeta potential result of the mixture of Ag-NPs and GO was − 7.1 mV, which potentially means that during the action of the ultrasounds, the GO flakes were coated by poly(vinyl alcohol) and Ag-NPs. The obtained zeta potential result of the mixture of Ag-NPs and GO sample in the water suspension implied that electrostatic stability was not present.

**Table 3:** Test results of zeta potential

Sample	ZP by LDE [mV] ± SD
Ag-NPs (100 μg/mL, after US)	− 5.9 ± 0,4
GO (200 μg/mL, after US)	− 41.0 ± 3
GO (200 μg/mL)-Ag (100 μg/mL, after US)	− 7.1 ± 0,5

*ZP* zeta potential, *LDE* laser Doppler electrophoresis, *SD* standard deviation, *US* ultrasound method, *Ag-NPs (as received)* HydroSilver1000, Amepox, Poland, material was not processed Ag-NPs (100 μg/mL, after US) as above but processed under US under similar conditions to the foils in the preceding sections, *GO (as received)* material not processed, *GO (200 μg/ml, after US)* as above but processed under US under similar conditions to the foils in the preceding sections, *GO (200 μg/mL)-Ag (100 μg/mL)* materials mixed together and processed under US under similar conditions to the foils in the preceding sections

### Foil Characteristics

**Fig. 7:**
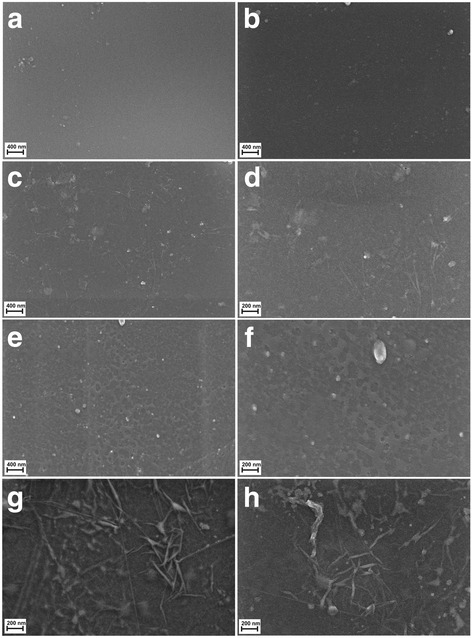
Scanning electron microscopy images of **a**, **b** non-coated polyurethane foil with a smooth surface with single impurities; **c**, **d** foil coated with GO, the broken-down GO flakes deposited on the polyurethane foil surface; **e**, **f** foil coated with Ag-NPs on which grid structures composed of polyvinyl alcohol and Ag-NPs are observed; and **g**, **h** foil coated with GO-Ag, which was mixed under the influence of ultrasounds and deposited on the foil surface

In order to determine the morphology of the created layers, four types of foil samples were compared (Fig. 7): pure polyurethane foil (A, B), GO-coated polyurethane foil (C, D), Ag-NP-coated polyurethane foil (E, F), and GO-Ag mixture-coated polyurethane foil (G, H).

Figure 7a, b shows an uncoated polyurethane foil with a smooth surface with single impurities. In Fig. 4c, d, the broken-down GO flakes deposited on the polyurethane foil surface are noticeable. Figure 7e, f shows the foil surface coated with Ag-NPs on which grid structures composed of polyvinyl alcohol and Ag-NPs are observable. Figure 7g, h presents a mixture of GO-Ag composition, which was mixed under the influence of ultrasounds and deposited on the foil surface.

### AFM Analysis and Surface Free Energy

**Fig. 8:**
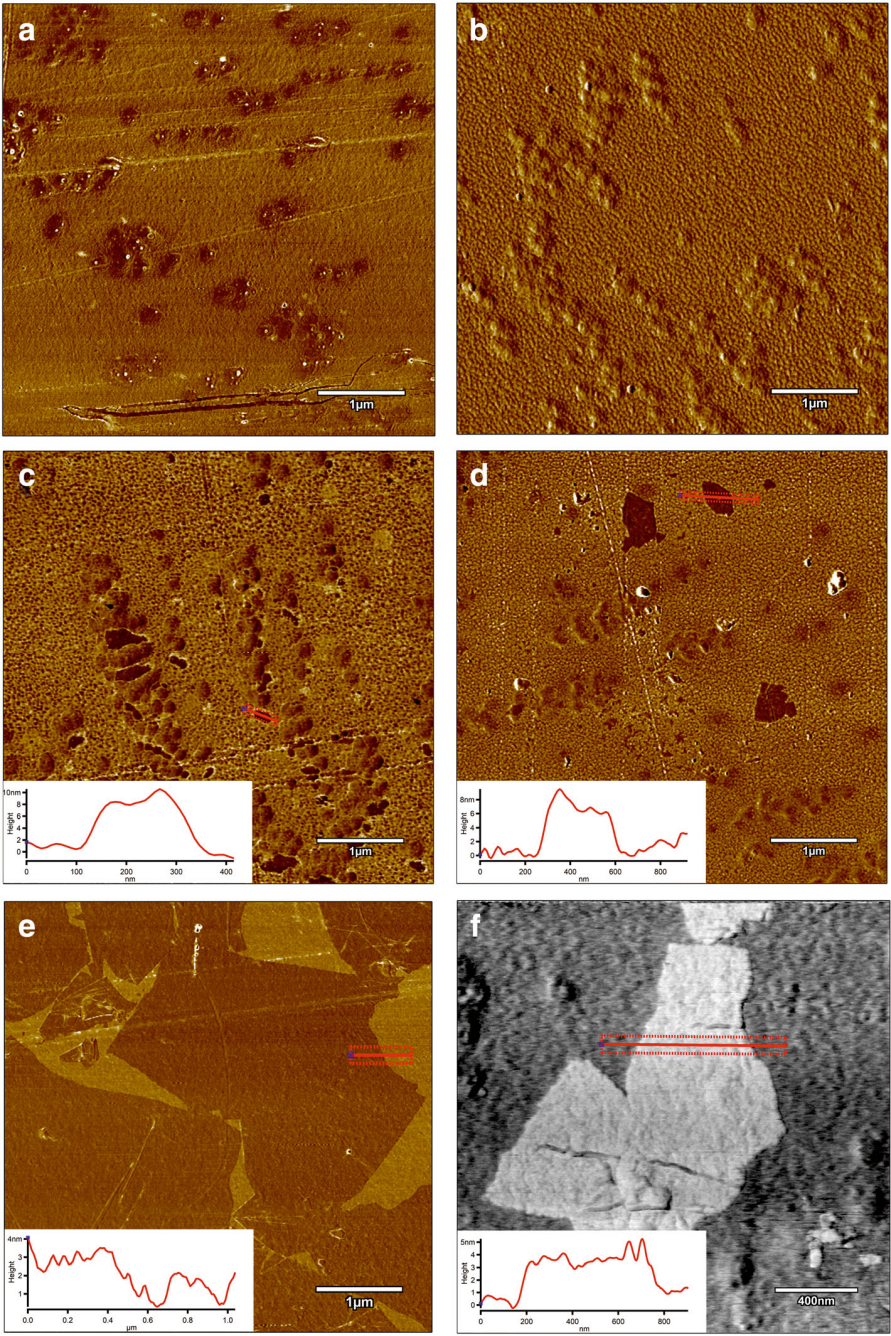
AFM phase contrast images and cross sections topographic images of graphene flakes: **a** non-coated foil polyurethane foil; **b** Ag-NPs coated foil where characteristic and homogeneous grid structures are observed; **c**, **d** GO-Ag-coated foil; **e** GO-coated foil, the surface of the foil almost entirely covered with GO flakes; the phase contrast image helps to observe these two phases, the darker area is GO and the lighter area is polymer foil; **f** LFM image of graphene flake. Red marks, area of cross section

AFM and LFM were used to complement the information about the surface morphology investigated by SEM. The investigation confirmed evolution of surface morphology by sonication of the polyvinyl alcohol with Ag-NP, GO, and GO-Ag NP solutions on the foils. Pure polyurethane foil was used as a reference foil in relation to foils coated by the ultrasonic method. The images in Fig. 8 are the AFM phase contrast images made in AC; additionally, cross sections of the GO flakes are attached under the corresponding images. Figure 8a is an image of pure polyurethane film; Fig. 7b depicts the Ag-NP-coated film, where characteristic and homogeneous grid structures are observable, being similar to those in Fig. 8e, f. Figure 8c, d shows GO-Ag-NP-coated film. Figure 8e shows the surface of the foil almost entirely covered with GO flakes; the phase contrast image helps to observe these two phases, the darker area is GO and the lighter area is polymer foil. It was noticed that the morphology of the foils has changed after Ag-NP coating comparing to the not-coated foils. The GO-Ag-NP coating differs from the previous one because it contains also small amounts of GO flakes seen as small black spots on the image, as it was mentioned earlier. Figure 8f depicts magnification of one GO flake made in LFM. The reduced friction confirms that it is, in fact, a GO flake.

The polar component for the GO-coated foil increased in relation to pure foil, from 2.3 ± 0.6 to 68.9 ± 2.8 mJ/m^2^, while the dispersion component decreased from 34.4 ± 1.3 to 8.2 ± 1.2 mJ/m^2^. SFE increased from 36.7 ± 1.4 to 77.0 ± 3.4 mJ/m^2^. A similar effect was not observed on foil surfaces coated with Ag-NPs and GO-Ag mixture. SFE of foil samples coated with Ag-NPs and GO-Ag mixture does not differ statistically (Table 4).

**Table 4:** Surface free energy of coated samples

Foil type	Dispersive [mJ/m^2^]	Polar [mJ/m^2^]	SFE [mJ/m^2^]
Foil (non-coated)	34.4	2.3	36.7
Foil + GO (200 μg/mL)	8.2	68.9	77.0
Foil + Ag-NPs (100 μg/mL)	30.0	11.3	41.3
Foil + GO (200 μg/mL)-Ag (100 μg/mL)	30.2	11.9	41.3

*Foil (non-coated)* polyurethane foil, *Foil + GO* polyurethane foil coated with graphene oxide, *Foil + Ag-NPs* polyurethane foil coated with silver nanoparticles, *Foil + GO-Ag* polyurethane foil coated with graphene oxide-based nanocomposite decorated with silver nanoparticles, *SFE* surface free energy

### Antibacterial Properties

**Fig. 9:**
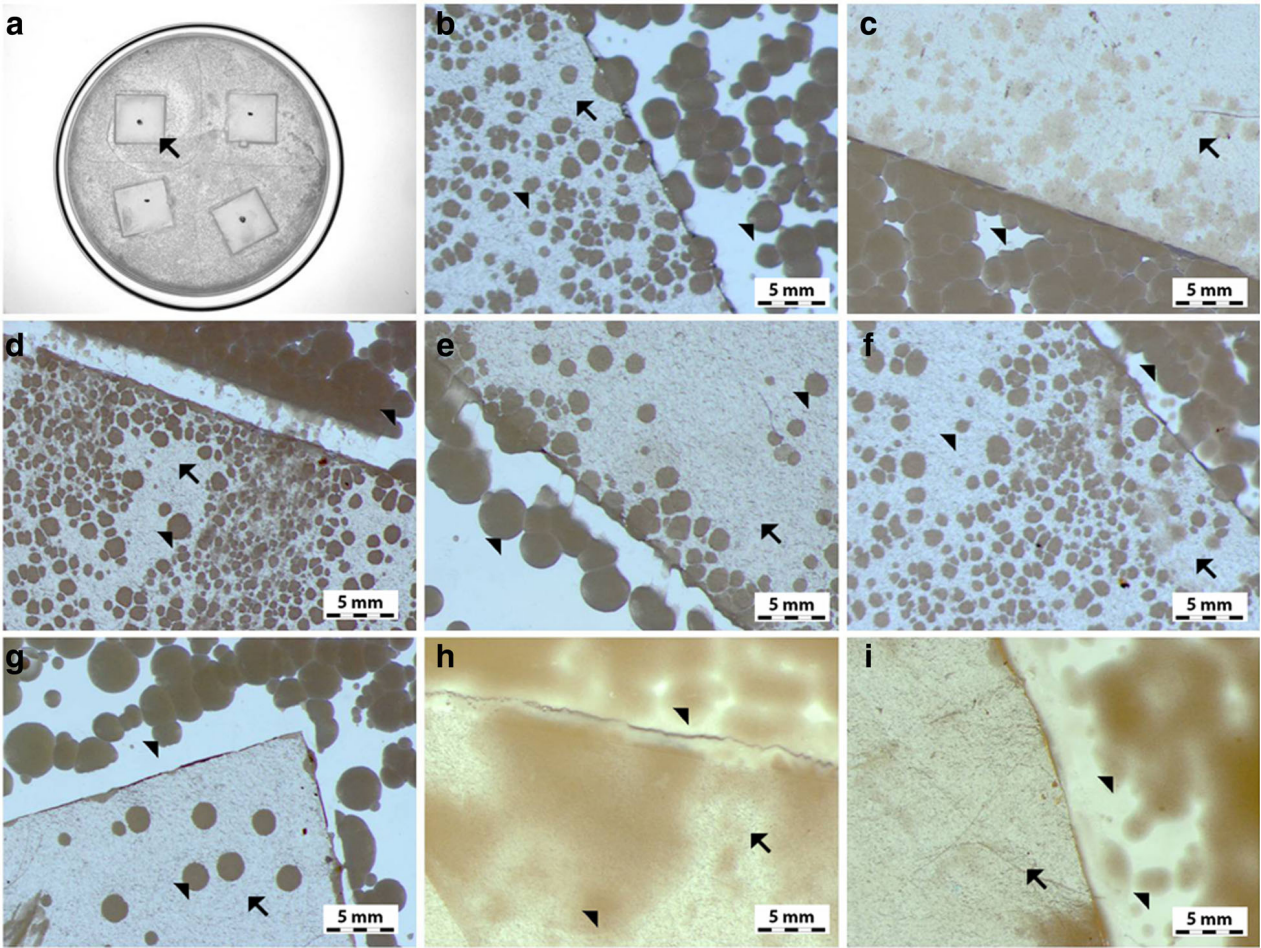
Antimicrobial properties of GO-Ag coated foils. The growth of *E*. *coli* (**b**, **c**), *S*. *aureus* (**c**, **d**), *S*. *epidermidis* (**e**, **f**), and *C*. *albicans* (**g**–**i**) colonies is reduced after co-incubation with GO-Ag-coated foils at 37 °C for 24 h. **a** Representative agar plate with GO-Ag-coated foils. Notes: Black arrows point to GO-Ag coated foils; arrowheads point to colonies of microorganisms

**Fig. 10:**
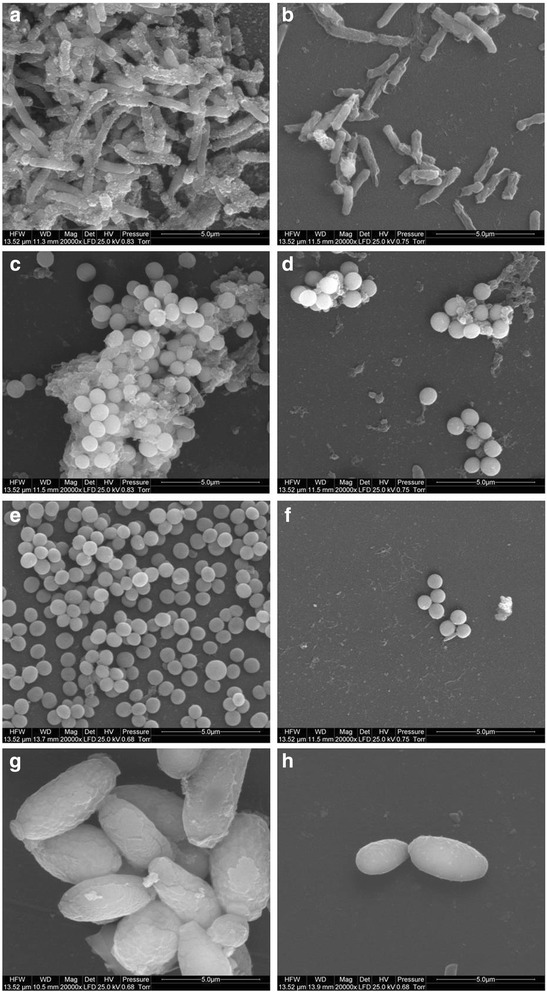
Morphology of microorganisms after co-incubation with GO-Ag-coated foils. Scanning electron microscopy images of bacteria and yeast in the control foils (**a**, **c**, **e**, **g**) and foils coated with GO-Ag (**b**, **d**, **f**, **h**) after incubation at 37 °C for 24 h. *E*. *coli* (**a**, **b**), *S*. *aureus* (**c**, **d**), *S*. *epidermidis* (**e**, **f**), and *C*. *albicans* (**g**, **h**) show decreased growth and deformed morphology after co-incubation with GO-Ag-coated foils

**Fig. 11:**
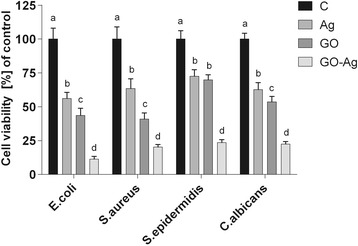
Foils coated with nanomaterials decrease *E*. *coli*, *S*. *aureus*, *S*. *epidermidis*, and *C*. *albicans* viability. Viability of bacteria and yeast after incubation on foils coated with Ag-NPs, GO, and GO-Ag at 37 °C for 24 h was assessed with Presto Blue assay. C control group (foil without nanoparticles), Ag foil coated with silver nanoparticles, GO foil coated with graphene oxide, GO-Ag foil coated with composite of graphene oxide and silver nanoparticles. Note: The columns with different letters (a–d) indicate significant differences between the concentrations (*P* = 0.001); error bars are standard deviations

The antibacterial activity of the different foils coated with GO, Ag-NPs, and GO-Ag were tested with *E*. *coli*, *S*. *aureus*, *S*. *epidermidis*, and *C*. *albicans.* Results showed that after co-incubation with bacteria at 37 °C for 24 h, foils inhibited the growth of all tested microorganisms but to various degrees. The maximum antibacterial effect against all tested microorganisms was with foil coated with the GO-Ag nanocomposite. The bacterial growth of the cells treated with foils coated with GO and Ag-NPs was slightly lower than that of cells in the control group whereas the growth of bacterial cells treated with foils coated with GO-Ag was greatly inhibited, 88.6% of *E*. *coli*, 79.6% of *S*. *aureus*, 76.5% of *S*. *epidermidis*, and 77.5% of *C*. *albicans* (Figs. 9, 10, and 11).

### Membrane Integrity

**Fig. 12:**
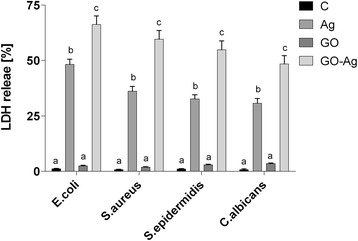
Foils coated with nanomaterials decreased *E*. *coli*, *S*. *aureus*, *S*. *epidermidis*, and *C*. *albicans* membrane integrity. Membrane integrity of bacteria and yeast after incubation on foils coated with Ag-NPs, GO, and GO-Ag at 37 °C for 24 h was assessed with LDH assay. C control group (foil without nanoparticles), Ag foil coated with silver nanoparticles, GO foil coated with graphene oxide, GO-Ag foil coated with composite of graphene oxide and silver nanoparticles. Note: The columns with different letters (a–c) indicate significant differences between the concentrations (*P* = 0.000); error bars are standard deviations

In cases where the cell membrane is damaged, intracellular LDH molecules could be released into the culture medium. The LDH level outside the cells demonstrates the cell membrane integrity. Foils coated with GO, Ag-NPs, and GO-Ag disrupted cell membrane functionality and integrity with significant differences between control groups and the Ag-NPs and GO-Ag groups (Fig. 12). The highest disruption of cell membranes was observed in the GO-Ag groups, 66.3% of *E*. *coli*, 59.4% of *S*. *aureus*, 54.8% of *S*. *epidermidis*, and 48.5% of *C*. *albicans*.

### ROS Production

**Fig. 13:**
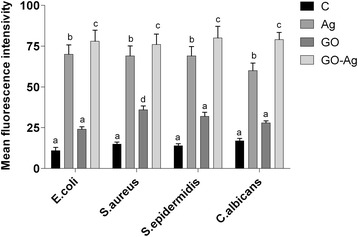
Effect of foils coated with nanomaterials on the production of reactive oxygen species. *E*. *coli*, *S*. *aureus*, *S*. *epidermidis*, and *C*. *albicans* were cultured on foils coated with Ag-NPs, GO, and GO-Ag at 37 °C for 24 h. Production of reactive oxygen species was assessed with DCFDA, Cellular Reactive Oxygen Species Detection Assay Kit. C control group (foil without nanoparticles), Ag foil coated with silver nanoparticles, GO foil coated with graphene oxide, GO-Ag foil coated with composite of graphene oxide and silver nanoparticles. Note: The columns with different letters (a–d) indicate significant differences between the concentrations (*P* = 0.000); error bars are standard deviations

Low levels (or optimum levels) of ROS play an important role in signaling pathways. However, when ROS production increases and overwhelms the cellular antioxidant capacity, it can induce macromolecular damage (by reacting with DNA, proteins, and lipids) and disrupt thiol redox circuits. Foils coated with Ag-NPs and GO-Ag (*P* < 0.05) increased the ROS production of all tested microorganisms compared to the control group. Foils coated with GO only increased the ROS production of *C*. *albicans*. The highest ROS production was observed in the GO-Ag group (Fig. 13).

## Discussion

The discovery of antibiotics, natural products produced by microorganisms that are able to prevent the growth of bacteria and thus cure infectious diseases, revolutionized medical therapy; however, the overuse and misuse of antibiotics have been key factors contributing to antibiotic resistance. Now, the era of antibiotics is coming to an end, and new antibacterial agents are needed. In recent years, studies have reported nanoparticles as a promising alternative to antibacterial reagents because of their antibacterial activity in several biomedical applications [19, 45]. Nanoparticles can be an effective way to control many pathogenic and antibiotic-resistant microorganisms. Among many metal nanoparticles, Ag-NPs have been intensely studied because of the distinct properties of their antibacterial activity [7]. Ag-NPs have been proved effective against over 650 microorganisms including bacteria (both gram-positive and negative), fungi, and viruses; however, the precise mechanism of antimicrobial action is not understood completely [46]. Ag-NP exposure to microorganisms could cause adhesion of nanoparticles to the peptidoglycan and the cell membrane [47], penetration inside the cell [48], induction of ROS [49], and damaging of intracellular structures [50]. However, bare Ag-NPs aggregate when they come into contact with bacteria; thus, they lose their active surface area and show weaker antibacterial activity [51]. To overcome this problem, nanocomposites composed of graphenic materials and Ag-NPs or other metal nanoparticles could be fabricated. GO with oxygen-containing functional groups is water soluble and therefore more biocompatible than pristine graphene. As a result, Ag-based GO nanocomposites may be used as antibacterial agents. However, the information about antimicrobial properties of graphene-based composites is limited, and mechanisms of toxicity or lack of toxicity are not fully explained.

The aim of this work was to study the action of graphene oxide-based nanocomposites decorated with Ag nanoparticles on *S*. *aureus*, *S*. *epidermidis*, *E*. *coli*, and *C*. *albicans* growth; membrane integrity; and ROS production. After co-incubation with the bacterial and yeast cells for 24 h, foils coated with GO-Ag nanocomposite inhibited the growth of all tested microorganisms with varying degrees, 88.6% of *E*. *coli*, 79.6% of *S*. *aureus*, 76.5% of *S*. *epidermidis*, and 77.5% of *C*. *albicans*. This action is most probably due to an increase in cell membrane and wall penetration by the nanoparticles. Some researchers suggest that the antimicrobial activity of graphene-based nanocomposites may be due to the disruption of cell membrane integrity and oxidative stress [52].

Foils coated with GO, Ag-NPs, and GO-Ag disrupted cell membrane functionality and integrity with significant differences between the control group and the Ag-NPs and GO-Ag groups. The highest disruption of cell membranes was observed in the GO-Ag groups, 66.3% of *E*. *coli*, 59.4% of *S*. *aureus*, 54.8% of *S*. *epidermidis*, and 48.5% of *C*. *albicans*. However, foils coated with bare Ag-NPs also disrupted cell membranes. It has been proposed that Ag-NPs are able to interact with bacterial membranes by increasing permeability and changing the structure of membranes, which finally leads to cell death [50]. Ag-NPs can cause direct damage to the bacterial cell membrane. Bacteria may be killed by the combined bactericidal effects of the released Ag^+^ ions and Ag nanoparticles. Additionally, the antimicrobial potential of Ag-NPs is also influenced by the thickness of the cell wall of the microorganisms [53]. The wall of gram-positive cells contains a thick layer (20–80 nm) of peptidoglycan, which is attached to teichoic acids. In gram-negative bacteria, the cell wall comprises a thin (7–8 nm) peptidoglycan layer and contains an outer membrane. The thicker peptidoglycan layer in gram-positive bacteria, such as *S*. *aureus* and *S*. *epidermidis*, may explain why these bacteria are more resistant to the antibacterial effects of GO-Ag.

Many studies have sought to establish a mechanism of action of antibacterial activity exhibited by silver in both its colloidal and ionic form. A disruption of membrane functionality from an interaction between released Ag^+^ ions and the cell membrane and extensive cell membrane damage caused by the formation of ROS ultimately causes damage to the cell due to oxidative stress. Additionally, Ag^+^ ions could cause dysfunction of the respiratory electron transport chain by uncoupling it from oxidative phosphorylation by inhibiting respiratory chain enzymes [54]. Foils coated with Ag-NPs and GO-Ag increased the ROS production of all tested microorganisms compared to the control group. The biological targets are DNA, RNA, proteins, and lipids. Lipids are one major target during oxidative stress. Free radicals can directly attack polyunsaturated fatty acids in bacterial and yeast membranes and activate peroxidation of lipids. A fundamental effect of lipid peroxidation is a decrease in membrane fluidity, which can significantly disrupt membrane-bound proteins. DNA is also a main target. Mechanisms of DNA damage involve abstractions and addition reactions by free radicals leading to carbon-centered sugar radicals and OH- or H-adduct radicals of heterocyclic bases. The sugar moieties producing single- and double-strand breaks in the backbone, adducts of base and sugar groups, and cross-links to other molecules can block replication. Foils coated with GO increased the ROS production at very low levels. However, Hu et al. [55] demonstrated that GO had a detrimental effect on *E*. *coli* due to decreased production of ATP and increased ROS production. Zhao et al. [56] reported the antibacterial activity of GO and reduced GO. Also, Gurunathan et al. [57] presented that GO and reduced GO showed significant antibacterial activity in a concentration- and time-dependent manner. Their results demonstrated that oxidative stress is a key mechanism for the antibacterial activity of GO and reduced GO through ROS generation. Nanda et al. [53] reported the effect of cystamine-conjugated GO against *E*. *coli*, *S*. *typhimurium*, *E*. *faecalis*, and *B*. *subtilis* with ROS production and high antibacterial activity.

Kurantowicz et al. [20] confirmed that bacteria could adhere to the GO surface, which results in the highest antibacterial activity. GO is characterized by a high degree of oxygenated functional groups: carbonyl, carboxylate, and hydroxyl. We hypothesize that these groups can be attractive groups for bacterial and yeast attachment. These groups are present on a large range of nutrients (amino acids, fatty acids) which are commonly recognized by microorganisms. In the present study, foils coated with GO induced membrane disruption and ROS production at a lower level than the Ag-NP and Ag-GO groups; however, cell viability was decreased, which is likely connected to the smaller active surface of GO after ultrasonic modifications.

## Conclusions

Ag-NPs, GO, and Ag-GO nanocomposites demonstrated the antibacterial activity that is stronger against gram-negative bacteria (*E*. *coli*) versus gram-positive bacteria (*S*. *aureus* and *S*. *epidermidis*) and yeast (*C*. *albicans*). The results showed that the decoration of GO with Ag-NPs promotes a synergistic effect and reduces dramatically the concentrations required to inhibit all tested bacterial and yeast strains. The antimicrobial potential of Ag-GO is also influenced by the thickness of the cell wall of the microorganisms. The thicker peptidoglycan layer in gram-positive bacteria, such as *S*. *aureus* and *S*. *epidermidis*, may explain why these bacteria are more resistant to the antibacterial effects of GO-Ag. A disruption of membrane functionality from an interaction between released Ag nanoparticles/Ag^+^ ions and the cell membrane and extensive cell membrane damage caused by the formation of ROS ultimately caused damage to the cell due to oxidative stress. In order to explain the mechanism of ROS production, additional studies are needed. Our research indicates the potential applicability of GO-Ag as an antimicrobial agent.

## Abbreviations

AFM: Atomic force microscopy
Ag-NPs: Silver nanoparticles
DLS: Dynamic light scattering
GO: Graphene oxide
GO-Ag: Graphene oxide decorated with silver nanoparticles
LDE: Laser Doppler electrophoresis
LDH: Lactate dehydrogenase
ROS: Reactive oxygen species
SEM: Scanning electron microscopy
XRD: X-ray diffraction

## References

[1] Jang S (2016). Multidrug efflux pumps in Staphylococcus aureus and their clinical implications. Journal of Microbiology (Seoul, Korea).

[2] Mathur P, Singh S (2013). Multidrug resistance in bacteria: a serious patient safety challenge for India. Journal of Laboratory Physicians.

[3] Sedighi M, Vaez H, Moghoofeie M (2015). Molecular detection of metallo-beta-lactamase gene blaVIM-1 in imipenem-resistant Pseudomonas aeruginosa strains isolated from hospitalized patients in the hospitals of Isfahan. Advanced Biomedical Research.

[4] Huh AJ, Kwon YJ (2011). “Nanoantibiotics”: a new paradigm for treating infectious diseases using nanomaterials in the antibiotics resistant era. Journal of Controlled Release.

[5] Lara HH, Garza-Trevino EN, Ixtepan-Turrent L, Singh DK (2011). Silver nanoparticles are broad-spectrum bactericidal and virucidal compounds. Journal of Nanobiotechnology.

[6] Chwalibog A, Sawosz E, Hotowy A (2010). Visualization of interaction between inorganic nanoparticles and bacteria or fungi. Int J Nanomedicine.

[7] Dar MA, Ingle A, Rai M (2013). Enhanced antimicrobial activity of silver nanoparticles synthesized by Cryphonectria sp. evaluated singly and in combination with antibiotics. Nanomedicine.

[8] Perelshtein I, Applerot G, Perkas N (2009). CuO–cotton nanocomposite: formation, morphology, and antibacterial activity. Surface and Coatings Technology.

[9] Wang L, Hu C, Shao L (2017). The antimicrobial activity of nanoparticles: present situation and prospects for the future. Int J Nanomedicine.

[10] Nagy A, Harrison A, Sabbani S (2011). Silver nanoparticles embedded in zeolite membranes: release of silver ions and mechanism of antibacterial action. Int J Nanomedicine.

[11] Salvioni L, Galbiati E, Collico V (2017). Negatively charged silver nanoparticles with potent antibacterial activity and reduced toxicity for pharmaceutical preparations. Int J Nanomedicine.

[12] Ghosh S, Patil S, Ahire M (2012). Synthesis of silver nanoparticles using Dioscorea bulbifera tuber extract and evaluation of its synergistic potential in combination with antimicrobial agents. Int J Nanomedicine.

[13] Fu PP, Xia Q, Hwang H-M (2014). Mechanisms of nanotoxicity: generation of reactive oxygen species. J Food Drug Anal.

[14] Huang C-C, Aronstam RS, Chen D-R, Huang Y-W (2010). Oxidative stress, calcium homeostasis, and altered gene expression in human lung epithelial cells exposed to ZnO nanoparticles. Toxicol in Vitro.

[15] Zhu X, Radovic-Moreno AF, Wu J (2014). Nanomedicine in the management of microbial infection—overview and perspectives. Nano Today.

[16] Seganish WM, Fischmann TO, Sherborne B (2015). Discovery and structure enabled synthesis of 2,6-diaminopyrimidin-4-one IRAK4 inhibitors. ACS Med Chem Lett.

[17] Soldano C, Mahmood A, Dujardin E (2010). Production, properties and potential of graphene. Carbon.

[18] Jaworski S, Sawosz E, Kutwin M (2015). In vitro and in vivo effects of graphene oxide and reduced graphene oxide on glioblastoma. Int J Nanomedicine.

[19] Masadeh MM, Karasneh GA, Al-Akhras MA (2015). Cerium oxide and iron oxide nanoparticles abolish the antibacterial activity of ciprofloxacin against gram positive and gram negative biofilm bacteria. Cytotechnology.

[20] Kurantowicz N, Sawosz E, Jaworski S (2015). Interaction of graphene family materials with Listeria monocytogenes and Salmonella enterica. Nanoscale Res Lett.

[21] Akhavan O, Ghaderi E (2010). Toxicity of graphene and graphene oxide nanowalls against bacteria. ACS Nano.

[22] Seil JT, Webster TJ (2012). Antimicrobial applications of nanotechnology: methods and literature. Int J Nanomedicine.

[23] Pelgrift RY, Friedman AJ (2013). Nanotechnology as a therapeutic tool to combat microbial resistance. Adv Drug Deliv Rev.

[24] Halouane F, Oz Y, Meziane D (2017). Magnetic reduced graphene oxide loaded hydrogels: highly versatile and efficient adsorbents for dyes and selective Cr(VI) ions removal. J Colloid Interface Sci.

[25] Applerot G, Perkas N, Amirian G (2009). Coating of glass with ZnO via ultrasonic irradiation and a study of its antibacterial properties. Appl Surf Sci.

[26] Perelshtein I, Applerot G, Perkas N (2010). Ultrasound radiation as a “throwing stones” technique for the production of antibacterial nanocomposite textiles. ACS Appl Mater Interfaces.

[27] Liu K-G, Abbasi AR, Azadbakht A (2017). Deposition of silver nanoparticles on polyester fiber under ultrasound irradiations. Ultrason Sonochem.

[28] Cierech M, Kolenda A, Grudniak AM (2016). Significance of polymethylmethacrylate (PMMA) modification by zinc oxide nanoparticles for fungal biofilm formation. Int J Pharm.

[29] Sivakumar M, Tang SY, Tan KW (2014). Cavitation technology—a greener processing technique for the generation of pharmaceutical nanoemulsions. Ultrason Sonochem.

[30] Moholkar V, Sivasankar T, Nalajala V (2011) Mechanistic aspects of ultrasound-enhanced physical and chemical processes. Handbook on Applications of Ultrasound. 10.1201/b11012-21.

[31] Gedanken A (2004). Using sonochemistry for the fabrication of nanomaterials. Ultrason Sonochem.

[32] Didenko YT, Suslick KS (2002). The energy efficiency of formation of photons, radicals and ions during single-bubble cavitation. Nature.

[33] Pokhrel N, Vabbina PK, Pala N (2016). Sonochemistry: science and engineering. Ultrason Sonochem.

[34] Chowdhury P, Viraraghavan T (2009). Sonochemical degradation of chlorinated organic compounds, phenolic compounds and organic dyes—a review. Sci Total Environ.

[35] Tzanakis I, Eskin DG, Georgoulas A, Fytanidis DK (2014). Incubation pit analysis and calculation of the hydrodynamic impact pressure from the implosion of an acoustic cavitation bubble. Ultrason Sonochem.

[36] Friedman A, Perkas N, Koltypin Y, Gedanken A (2012). Depositing nanoparticles inside millimeter-size hollow tubing. Appl Surf Sci.

[37] Goncalves G, Vila M, Bdikin I (2014). Breakdown into nanoscale of graphene oxide: confined hot spot atomic reduction and fragmentation. Sci Rep.

[38] Liscio A, Kouroupis-Agalou K, Betriu XD, et al (2017) Evolution of the size and shape of 2D nanosheets during ultrasonic fragmentation. 2D Materials 4:025017. 10.1088/2053-1583/aa57ff.

[39] Poh HL, Sanek F, Ambrosi A (2012). Graphenes prepared by Staudenmaier, Hofmann and Hummers methods with consequent thermal exfoliation exhibit very different electrochemical properties. Nano.

[40] Wilson AJM, Phillips M, Wilson NR (2013). Friction force microscopy: a simple technique for identifying graphene on rough substrates and mapping the orientation of graphene grains on copper. Nanotechnology.

[41] Żenkiewicz M (2007). Comparative study on the surface free energy of a solid calculated by different methods. Polym Test.

[42] Ferrari AC (2007). Raman spectroscopy of graphene and graphite: disorder, electron–phonon coupling, doping and nonadiabatic effects. Solid State Commun.

[43] Pérez-Martínez P, Galvan-Miyoshi JM, Ortiz-López J (2016). Ultrasonic cavitation effects on the structure of graphene oxide in aqueous suspension. J Mater Sci.

[44] Stobinski L, Lesiak B, Malolepszy A (2014). Graphene oxide and reduced graphene oxide studied by the XRD, TEM and electron spectroscopy methods. J Electron Spectrosc Relat Phenom.

[45] Singh P, Kim YJ, Singh H (2015). Biosynthesis, characterization, and antimicrobial applications of silver nanoparticles. Int J Nanomedicine.

[46] Dakal TC, Kumar A, Majumdar RS, Yadav V (2016). Mechanistic basis of antimicrobial actions of silver nanoparticles. Front Microbiol.

[47] Sondi I, Salopek-Sondi B (2004). Silver nanoparticles as antimicrobial agent: a case study on E. coli as a model for Gram-negative bacteria. J Colloid Interface Sci.

[48] Schreurs WJ, Rosenberg H (1982). Effect of silver ions on transport and retention of phosphate by Escherichia coli. J Bacteriol.

[49] Pellieux C, Dewilde A, Pierlot C, Aubry JM (2000). Bactericidal and virucidal activities of singlet oxygen generated by thermolysis of naphthalene endoperoxides. Methods Enzymol.

[50] Lok C-N, Ho C-M, Chen R (2006). Proteomic analysis of the mode of antibacterial action of silver nanoparticles. J Proteome Res.

[51] Hajipour MJ, Fromm KM, Ashkarran AA (2012). Antibacterial properties of nanoparticles. Trends Biotechnol.

[52] Quinteros MA, Cano Aristizabal V, Dalmasso PR (2016). Oxidative stress generation of silver nanoparticles in three bacterial genera and its relationship with the antimicrobial activity. Toxicol in Vitro.

[53] Nanda SS, An SSA, Yi DK (2015). Oxidative stress and antibacterial properties of a graphene oxide-cystamine nanohybrid. Int J Nanomedicine.

[54] Belluco S, Losasso C, Patuzzi I (2016). Silver as antibacterial toward Listeria monocytogenes. Front Microbiol.

[55] Hu W, Peng C, Luo W (2010). Graphene-based antibacterial paper. ACS Nano.

[56] Zhao J, Deng B, Lv M (2013). Graphene oxide-based antibacterial cotton fabrics. Advanced Healthcare Materials.

[57] Gurunathan S, Han JW, Dayem AA (2012). Oxidative stress-mediated antibacterial activity of graphene oxide and reduced graphene oxide in Pseudomonas aeruginosa. Int J Nanomedicine.

